# Evaluation of the HIV Case‐Based Surveillance System in Dire Dawa City Administration, Ethiopia. A Descriptive Cross‐Sectional Study

**DOI:** 10.1155/arat/6539109

**Published:** 2026-01-23

**Authors:** Fitsum Hagos, Aklesiya Kassahun, Ashenafi Sisay

**Affiliations:** ^1^ Public Health Emergency Management, Ethiopian Public Health Institute (EPHI), Addis Ababa, Ethiopia, ephi.gov.et

**Keywords:** case-based surveillance, Dire Dawa, Ethiopia, HIV, REDCap, surveillance evaluation

## Abstract

**Background:**

Ethiopia started the human immunodeficiency virus (HIV) case‐based surveillance (CBS) system along with Research Electronic Data Capture (REDCap) in June 2021. From January to June 2022, only five out of 14 CBS implementing health facilities in Dire Dawa City, Ethiopia, reported 35 newly diagnosed HIV patients through the REDCap Database compared to 314 in the District Health Information System (DHIS‐2). This study aimed to evaluate the CBS system, its usefulness, and reasons for underreporting in Dire Dawa City, Ethiopia.

**Methods:**

We used a descriptive cross‐sectional study design. We customized the data collection tools from the Centers for Disease Control and Prevention (CDC), a guideline for evaluating public health surveillance systems. Questionnaires were administered to 36 healthcare workers involved in supporting CBS. Completed HIV CBS case reporting forms were also assessed for completeness. EPI Info software was used for data entry and analysis. Descriptive statistics, such as frequencies and proportions, were used to describe the findings.

**Results:**

Interviews were successfully conducted with 34 health workers. The lack of CBS reporting guidelines for healthcare facilities was 22 (61%). Limited coordination between technical staff and health facilities 19 (53%) and limited competency in REDCap 23 (64%) were also observed. CBS data timeliness, completeness, and validity were 89%, 87%, and 99%, respectively, in the REDCap. There is a lack of standard operating procedures during system interruption. The overall health facility representativeness was 368 of 757 (49%). Acceptability was 100%, mainly due to reduced paperwork and the ability to generate simple reports.

**Conclusion:**

and Recommendations: The HIV CBS system was timely and acceptable. However, its representativeness was poor owing to limited competency in the REDCap. We recommend that health workers receive further training for case‐based HIV surveillance.

## 1. Introduction

The U.S. Department of Health released the HIV National Strategic Plan (NSP) in January 2021, aiming to reduce new HIV acquisitions by 90% by 2030, which was replaced by the National HIV/AIDS Strategy in December 2021 [1].

Ethiopia’s NSP for HIV outlines a comprehensive framework aimed at achieving sustained epidemic control. Its vision is to realize an AIDS‐free Ethiopia, while the mission focuses on instituting effective HIV prevention and control programs, coordinating the national response, and strengthening health systems and enabling environments. The NSP’s overarching goal is to attain and sustain HIV epidemic control by 2027 by reducing both new HIV infections and AIDS‐related mortality to fewer than 1 per 10,000 population nationally and across all subnational and key population groups. The strategy prioritizes measurable impact targets, including reducing new HIV infections and HIV‐related deaths to below 1 per 10,000 population, decreasing the incidence–mortality ratio to less than 1, and lowering the rate of mother‐to‐child transmission among HIV‐positive women from 12% in 2022 to below 5% by 2027. Implementation of the NSP is guided by key principles: a multisectoral approach that engages all sectors; inclusiveness and a people‐centered lens that supports diverse prevention options; strong community leadership in HIV response activities; gender responsiveness to address the needs of women, men, girls, and boys; and a value‐for‐money framework emphasizing equity, efficiency, effectiveness, and sustainability throughout program design, execution, and evaluation. This strategic direction forms the basis for Ethiopia’s coordinated efforts to achieve HIV epidemic control [2].

However, existing HIV/AIDS information systems have several limitations. For instance, it does not distinguish whether the diagnosis of a new HIV case is due to an increase in HIV transmission or increased testing coverage for undiagnosed HIV acquisitions, lacks a real‐time data reporting system, or does not track individual‐level data. Strengthening national HIV/AIDS strategic information systems through longitudinal and individual‐level data is the World Health Organization’s (WHO) recommendation to its member states to better understand subnational epidemics and guide more focused interventions [3]. Ethiopia initiated the implementation of HIV case reporting and recency testing surveillance in June 2019 based on the technical guidelines of the Ministry of Health (EPHI) technical guideline. The system includes several major activities—such as routine HIV case reporting, HIV testing services (HTS) monitoring, ART service delivery tracking, viral load result management, and community‐based follow‐up—currently rolled out in more than 400 high‐volume public and private HIV health facilities and community drop‐in centers [1].

The objective of the evaluation of the HIV case‐based surveillance (CBS) system study aimed to evaluate the CBS system, its usefulness, and reasons for underreporting in Dire Dawa City, Ethiopia. The CBS system also aims to monitor and describe epidemiological patterns in newly diagnosed HIV cases based on demographics, behavior, method of transmission, and time after HIV acquisition. Furthermore, the system intends to track and report trends in clinical state (WHO stage, initial CD4 count, and other opportunistic acquisitions) at the time of diagnosis. The United States Centers for Disease Control and Prevention (CDC) emphasizes the need for routine evaluation of disease surveillance systems within a district, region, or nation [4]. The functionality of the surveillance system was determined through a surveillance system evaluation. Hence, the evaluation of a surveillance system is vital for identifying gaps in the system and ensuring improvement in the quality, efficiency, and usefulness of the system.

### 1.1. Flow of Data in the HIV CBS System in Ethiopia

**Figure 1:**
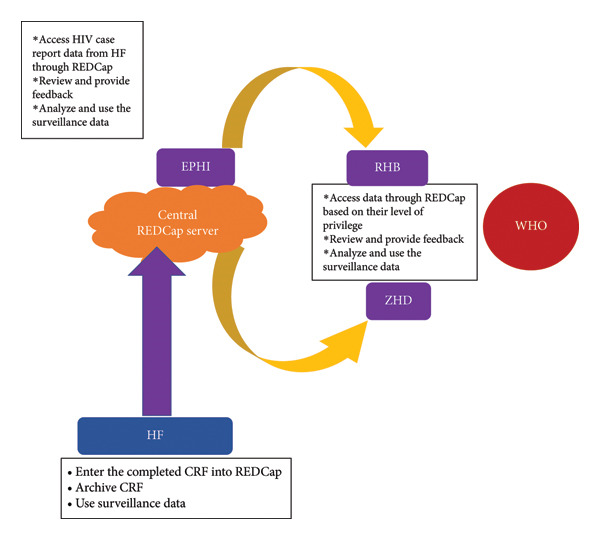
The HIV case‐based surveillance information flowchart in Ethiopia.

At the health facility level, HIV case reports are generated at the point of service delivery, such as the hospital outpatient department or inpatient department, Family and Child Health (FCH) unit, and Voluntary Male Medical Circumcision (VMMC) unit. At health facilities that use REDCap, all surveillance data elements are recorded on a single HIV case report form (CRF). The data from this CRF were then transferred to the REDCap system using an on‐site computer device. Data from both the e‐first and e‐last clinic‐based REDCap databases were merged into the national shared health record component through web‐based applications with secure internet connectivity. A routine process was used to remove duplicate health records across clinics by applying software that compares demographic variables. After deduplication and anonymization, individual health records were transmitted to the national server in the EPHI database through the shared health record component [5] (Figure 1). Fourteen health facilities in the Dire Dawa City Administration in eastern Ethiopia implemented CBS using the REDCap database in 2019 as part of broader efforts to reduce inefficiencies associated with paper‐based data collection and reporting. At these facilities, individual‐level information for all newly diagnosed people with HIV was collected using CBS forms and entered into REDCap, which served as the case‐based reporting platform and transmitted anonymized records to the national server. Although DHIS‐2 has the technical capacity to capture patient‐level data through its Tracker module, it is not used for case‐based HIV reporting in Ethiopia. Instead, DHIS‐2 functions primarily as an aggregate reporting system, where health facilities submit monthly summary totals of new HIV diagnoses compiled from HTS registers. Accordingly, while the systems differ in functionality—REDCap capturing detailed individual‐level case reports and DHIS‐2 providing aggregate monthly summaries—the total number of new HIV diagnoses reported through REDCap should correspond with the aggregate figures reported in DHIS‐2.

This study aimed to evaluate the CBS system, its usefulness, and reasons for underreporting in Dire Dawa City, Ethiopia [6]. The Dire Dawa City Administration was selected purposively from the nine regional states and two city administrations based on its relatively high HIV prevalence and the absence of previous evaluations of the HIV CBS system. The estimated adult HIV prevalence in Dire Dawa is 2.67%, which is higher than the national average of 1.54%; it is lower than in Gambela (4.52%) and Addis Ababa (3.52%), but substantially higher than in regions such as Somali (0.09%) and the Southern Nations, Nationalities, and Peoples’ Region (0.36%).

## 2. Methodology

### 2.1. Study Design

The study design used for this research was a descriptive cross‐sectional approach aligned with the updated guidelines provided by the CDC for evaluating public health surveillance systems [7]. In addition to quantitative analysis, qualitative methods were integral to this study. Qualitative techniques, including interviews, observations, and document reviews, were used to gain a comprehensive understanding of the nuances and contextual factors influencing the effectiveness of the surveillance systems under investigation. These qualitative methodologies enriched the analysis by providing insights into stakeholders’ perspectives, operational challenges, and potential areas for improvement within the public health surveillance framework [8].

### 2.2. Study Setting

Based on 2012 (EFY) figures from the Central Statistical Agency (CSA) of Ethiopia, Dire Dawa has an estimated total population of 506,639, consisting of 285,377 males and 221,262 females [9]. A total of 32.2% of the population was estimated to be rural inhabitants, while 67.5% were urban dwellers [10]. All health facilities offered HIV testing and counseling services; however, HIV CBS implemented health facilities were 14 at the time of the study [8]. We included in this study all 14 health facilities that had implemented HIV CBS. These were Sabiyan Primary Hospital, Dil Chora Hospital, Yemaryam Work General Hospital, Art General Hospital, Bilal Hospital, Goro Health Center, Gende Kore Health Center, Legehare Health Center, Gendegerada Health Center, Dechatu Health Center, Addis Ketema Health Center, Diredawa Health Center, FGA Health Clinic, and Melka Jebdu Health Centers.

### 2.3. Study Population

We interviewed healthcare workers involved in the HIV CBS system and HIV testing and counseling services as primary respondents. The Regional Health Bureau HIV CBS focal persons, health facility level focal persons, nurses in charge, REDCap data clerks in facilities, and provincial health information officers were interviewed as key informants. The study participants were RHBs, Woreda health offices, and health facilities (hospitals and health centers) found in the city administration.

### 2.4. Sample Size Determination

To assess the completeness of HIV CBS case reporting forms, we calculated a minimum sample size of 80 HIV CBS reporting forms using Dobson’s formula for calculating the sample size for a single population proportion:

(1) n=Zα/22p1−pc2,

where Z = Z statistic value that is 1.96 for a 95% confidence interval; *p* = proportion of HIV CBS reporting forms with 76% completeness; c = 10% precision.

Assume that the completeness of HIV CBS case reporting forms is 76% complete, given a 95% confidence interval, a 10% level of precision, and a 14% nonresponse rate.

(2) n 1.962p1−pc2n=1.9620.7610.76−0.12=701480∗%=.

## 3. Sampling Techniques

### 3.1. Sampling of Primary Participants

All of the health workers who were engaged in HIV testing and counseling, as well as data entry, were found to be on duty at the health facilities over the course of the study. In addition, we enrolled participants from the RHBs.

### 3.2. Sampling of HIV CBS Forms

We used the random sampling function in Microsoft Excel (” = RAND()”) to select seven HIV CBS forms from each health facility included in the study. First, a comprehensive line list of all HIV CBS reporting forms was created by extracting the monthly sequential numbers assigned in the HTS register. Each form was assigned a unique identifier within the spreadsheet. The Excel randomization function was then applied to generate a random number for each form, and the list was sorted in ascending order to select the required number of forms per facility. In total, 80 paper‐based HIV CBS case reporting forms collected between November 1, 2022, and October 30, 2023, were sampled. Each selected form was then reviewed for completeness, internal consistency, accuracy of recorded variables, and concordance with corresponding entries in the HTS register. These checks allowed us to assess the quality of data recorded on the forms using measurable indicators such as missing fields, discrepancies, and logical errors.

### 3.3. Data Collection

We used interviewer‐administered questionnaires to collect the demographic characteristics of healthcare workers participating in the study and information on the reasons for underreporting of newly diagnosed HIV patients in the REDCap system from healthcare workers and key informants, as well as to assess system attributes. Using standardized formats, we extracted secondary data from the surveillance reports.

### 3.4. Health Worker Knowledge of the HIV CBS System With Recency Testing

We used interviewer‐administered questionnaires to assess health workers’ knowledge of the HIV CBS system with recency testing using the following variables: ability to accurately define a case, ability to describe the purpose of the HIV CBS system, ability to state what information is collected from a case in the HIV CBS system, ability to accurately state which sentinel events are reportable, and ability to describe the steps taken after an HIV case is diagnosed. We used a 5‐point Likert scale to assess health worker knowledge levels [11]. Health workers who answered less than or equal to two questions correctly out of five were classified as having poor knowledge levels, and those who scored three out of five were classified as having fair knowledge levels. Participants who answered four or more questions correctly were classified as having good knowledge levels.

## 4. Evaluation of HIV CBS System Attributes

### 4.1. Representativeness

Representativeness measures the degree to which the CBS system captures all cases and sentinel events and the degree to which cases in the surveillance system are representative of all cases in the population. We assessed representativeness by examining the proportion of HIV cases reported in the REDCap system compared to those recorded in the DHIS‐2 using monthly reports. We measured representativeness as the (1) proportion of healthcare facilities in the city administration (private or public) that submit case reports to the national system and (2) proportion of HIV cases diagnosed in a quarter that were reported in the DHIS‐2 and CBS systems.

### 4.2. Timeliness

This is the time between the date of HIV diagnosis and its entry into the REDCap. All CRFs should be ready and entered within the specified time frame within 15 days, having been checked and verified. The percentage of timely reporting facilities in a given area shows which institutions report on time. We determined timeliness by assessing the lag time between diagnosis and entering the case report in the CBS system.

### 4.3. Data Quality

The data quality reflects the completeness and validity of the data recorded in the HIV CBS system. Completeness refers to the proportion of case reports containing all essential variables. We assessed completeness by calculating the proportion of case reports with all completed case‐defining variables. Responses marked as “unknown” or left blank were considered incomplete when applicable to the HIV case. Additionally, data quality was evaluated based on the number of trainings, supervision visits, and data quality assurance reviews conducted. Validity refers to the extent to which CBS data are logically and temporally consistent. We assessed validity by examining birth year, which was required to be a four‐digit number between 1900 and 2022, and by verifying that the HIV diagnosis date occurred after the client’s date of birth, as any diagnosis recorded prior to birth was considered invalid.

### 4.4. Simplicity

Simplicity refers to the ease of operation, structure, and integration of incidence and routine HIV surveillance. We assessed the simplicity of the HIV CBS system with recency testing using the proportion of health workers who found completing paper‐based and electronic forms to be easy and not time‐consuming (average time taken to complete these forms) and staff training requirements.

We assessed the surveillance system by testing its functionality across several key operations. Specifically, we evaluated the ability to log into the system, create an HIV electronic health record, retrieve a client’s record, edit or update an existing record, delete a record, and generate reports. Each function was performed by study personnel to determine whether the system could successfully support routine surveillance tasks.

### 4.5. Acceptability

Acceptability reflects the willingness of healthcare workers to participate in case‐based HIV surveillance. We assessed acceptability through interviews with health workers. Using data from the surveillance system, we determined the proportion of healthcare workers willing to continue participating. We objectively assessed the acceptability of the HIV CBS system using completeness, quality of data, timeliness, availability of meeting minutes, and feedback reports.

### 4.6. Flexibility

A flexible surveillance system can adapt to changing information needs or operating conditions with minimal additional time, personnel, or funds. We assessed the flexibility of the HIV CBS system by collecting information from health workers on its ability to integrate with other systems, adapt to new reporting requirements, and respond to emerging public health events such as COVID‐19, conflicts, and natural disasters. Specifically, we asked about system integration with DHIS‐2 and the Electronic Patient Monitoring System (EPMS), the need for additional staff or resources when new information was introduced, and the ease of sharing data through existing reporting platforms.

### 4.7. Stability

Stability refers to the reliability and availability of an HIV CBS system. We assessed the stability of the surveillance system by ascertaining the presence of dedicated staff for HIV CBS activities and the level of interruption of the system due to inadequate human resources, REDCap system downtime due to electricity outages, availability of CRFs, and dedicated computers to enter case reports using a checklist.

### 4.8. Usefulness

We assessed the usefulness of the HIV CBS system by asking respondents if the collected data were analyzed, the data used, and any reports or graphs generated from the data. The respondents were also asked about the public health actions carried out or made based on the findings from the data collected by the surveillance system. We also assessed the availability of minutes of meetings held on the surveillance system and any actions taken to validate its usefulness.

### 4.9. Data Analysis

We used Epi Info TM version 7.2.4 for data entry and analysis. Descriptive statistics were used to analyze the quantitative data and are presented as medians, interquartile ranges, frequencies, and proportions. A 5‐point Likert scale was used to rate the respondents’ knowledge.

## 5. Results

### 5.1. Demographic Characteristics of the Respondents

We interviewed 34 healthcare workers, the majority of whom were female (22/34, 65%) and diploma nurses (13/34, 38%). The median age of the study participants was 25.4 years (interquartile range [IQR]: 22.3–29.3), and the median years of service was 4 years (IQR: 6–10) (Table 1).

**Table 1:** Demographic characteristics of health workers involved in HIV CBS activities at REDCap implementing facilities in Dire Dawa, 2022.

Variable	Categories	Frequency *n* = 34	Percentage
Sex	Female	22	65
	Male	12	35
Designation	Nurse (diploma)	13	38
	Nurse (degree)	9	26
	Health officer (HO)	6	18
	Data entry clerks	6	18
Age group (years)	20—24	15	44
	25–29	11	32
	30—34	5	15
	35–39	3	9

The major reasons reported by health workers for underreporting newly diagnosed HIV‐positive cases through the REDCap database were work overload/understaffed 25/34 (73%), high staff turnover 22/34 (65%), and inadequate REDCap database competence 17/34 (50%). Limited coordination between units: 14/34 (41%); absence of feedback from higher levels: 11/34 (32%); and use of parallel systems: 9/34 (26%) (Table 2).

**Table 2:** Reasons for underreporting of newly diagnosed HIV cases through the REDCap database at health facilities in Dire Dawa, 2022.

Reason for underreporting	Frequency *n* = 34	Percentage
Work overload/short staffed	25	73
High staff turnover	22	65
Inadequate REDCap database competence	17	50
Limited coordination between units	14	41
No feedback from higher levels	11	32
Electricity power outages	10	29
Use of parallel systems for reporting cases	9	26
No reporting guidelines	8	22
Poorly designed interface	6	18
Too many data sources required to fill the form	5	15
Poor network connectivity	2	6

### 5.2. Health Worker Knowledge About the HIV CBS System in Dire Dawa, 2022

Of the 34 health workers assessed for awareness and understanding of the HIV CBS system, 31 (91%) were able to accurately define an HIV‐positive case, while 28 (82%) were able to describe the objectives of the HIV CBS system. A total of 33/34 (97%) knew which information was collected for HIV case reporting, and 33/34 (97%) were able to accurately state reportable sentinel events. Actions taken after an HIV‐positive case was diagnosed were accurately described by 31/34 (91%) healthcare workers. Using a 5‐point Likert scale, 28/34 (82%) healthcare workers had good knowledge, 4/34 (12%) had fair knowledge, and 2/34 (6%) had poor knowledge of the HIV CBS system (Table 3).

**Table 3:** Health worker knowledge levels of the HIV case‐based surveillance system at CBS implementing facilities in Dire Dawa, 2022.

Variable	Frequency *n* = 34	Percentage
Health workers who knew at least two (≥ 2/3) definitions of an HIV case	31	91
Health workers who knew at least two (≥ 2/4) objectives of the HIV CBS system	28	82
Health workers who knew at least eight (≥ 8/16) patients variables collected on an HIV case	33	97
Health workers who knew at least four (≥ 4/8) reportable sentinel events	33	97
Health workers who knew at least 2 (≥ 2/4) actions to be taken when an HIV case has been identified	31	91
Overall health worker knowledge levels of the HIV CBS system good (4–5 correct)	28	82
Fair (3 correct)	4	12
Poor (1–2 correct)	2	6

### 5.3. The HIV CBS System Attributes at CBS Implementing Facilities in Dire Dawa

#### 5.3.1. Representativeness

Compared to the DHIS‐2 system, 3/156 (2%) of HIV cases were reported through the CBS system from July to August 2019, which increased to 169/233 (72%) by April–June 2022. Overall, the representation was 44%. Three private facilities reported cases through the HIV CBS system; however, 31/36 (86%) of the healthcare workers reported that there were governmental organizations that performed community testing as well as outreach activities (FGA) clinics that reported HIV cases to the facilities through the HIV CBS system.

#### 5.3.2. Timeliness

Of the 34 health workers interviewed, 31 (91%) still used both the notification systems. At facilities using both paper‐based and electronic systems, 23/25 (92%) of healthcare workers entered the HIV CBS case reporting forms daily, and overall 96% of CRFs were entered within 15 days. Data transmission from the facility to the national level was automatic (real time) once entered, as reported by 31/34 (91%) of the healthcare workers (Table 4).

**Table 4:** Timeliness, simplicity, flexibility, acceptability, and usefulness of the HIV CBS system at CBS implementing facilities in Dire Dawa, 2022.

Variable	Frequency	Percentage
Timeliness of the HIV CBS system		
Still using both CBS case reporting forms and DHIS‐2	31	91
Time period taken for entry of paper‐based HIV CBS case reporting forms into the REDCap database (n = 25)		
Daily	23	92
Weekly	1	4
Monthly	1	4
Timely transmission of CBS data from the facility to the national level		
Yes	32	94
No	2	6
Simplicity of the CBS system		
Access the REDCap database	6	100
Generate a report	32	89
Edit or update data on the REDCap	29	80
Median time taken to complete HIV CBS case reporting form median 10 min (Q1=7)	(Q1 = 7)	(Q3 = 14)
Acceptability of the CBS system		
Health workers who reported a case in the CBS system	34	100
Health workers who completed an HIV CBS case reporting form (CRF)	33	97
Health workers willing to participate in the system	31	91
Health workers able to do data analysis and use	28	82
Flexibility of the HIV CBS system		
Ease of integration with the DHIS‐2/PHEM system	33	97
Ease of sharing CBS data through the PHEM reporting system	32	94
Ease of integrating new information requirements	30	88
More staff required to operate the system	28	82
Usefulness of the HIV CBS system		
Held meeting on CBS as an agenda in regular meeting	31	86
Data analysis performed at the facility level	23	68
Public health action taken	21	62
Trend line from HIV CBS data	20	59
Availability of minutes	18	53
Risk behaviors identified for hotspot areas	18	53

#### 5.3.3. Data Quality

Eighty CRFs were assessed, and the overall completeness was 84%. Data completeness ranged from as high as 95% for client identifier information and 88% for facility information to as low as 44% for the assessment of WHO clinical staging at diagnosis. Validity was 95% of the assessed 80 CRFs after the patients’ age was verified by comparing the age of the patient with the calculated age based on the date of birth. Thirty‐four healthcare workers (94%) reported that they had received a supervisory visit at a higher level. The median reported number of supervisory visits was three (IQR, 2–6) (Table 5).

**Table 5:** Data quality of the HIV case‐based surveillance system at facilities in Dire Dawa, 2022.

Variable	Frequency *N* = 80	Percentage	Completeness (%)
Section A: Client identifier information	80	100	95
Section B: Facility information	78	97	88
Section C: Index testing	75	94	85
Section D: Client demographic information	79	99	89
Rapid test for HIV recent acquisition (client aged 15 years and above)	75	94	81
ART initiation	77	96	89
Assessment of WHO clinical staging at notification	27	34	44
Validity	79	99	
Supervisory visits from higher levels	Median 3 Q1 = 2		Q3 = 6

#### 5.3.4. Simplicity

Of the 34 health workers interviewed, 32 (94%) reported that case reporting forms were not difficult to complete. The median time taken to complete one form was 10 min (IQR, 7–14) compared to 20 min (IQR, 8.5–29.5) when completing a case reporting form in the DHIS‐2 system. The use of the REDCap database ranged from 34/34 (100%) data clerks who were able to access the REDCap to 29/34 (85%) being able to edit or update an electronic health record (Table 4).

#### 5.3.5. Acceptability

All 36 (100%) healthcare workers reported their willingness to continue participating in the HIV CBS system and had previously reported a case, while 33/36 (92%) reported that they were part of a team that analyzed and utilized HIV CBS data (Table 4).

#### 5.3.6. Flexibility

Health workers reported that the CBS system was easy to integrate with DHIS‐2 (33/34, 97%), and most were able to share HIV CBS data through the PHEM reporting system, which included DHIS‐2 and EPMS (32/34, 94%). However, the integration of new information required additional staff for 28/34 (82%) of respondents, and 30/36 (83%) noted extra effort was needed to accommodate new data requirements.

#### 5.3.7. Stability

Healthcare workers dedicated to HIV CBS activities constituted 27/36 (75%) respondents. Of the 14 facilities assessed, seven did not have a generator, two had nonfunctioning generators and needed repairs, and two health facilities did not have solar power, which affected the operation of the CBS system. We found that these challenges were associated with fuel shortages for the generators. Eight facilities did not have WHO clinical staging of HIV disease guidelines.

#### 5.3.8. Usefulness of the HIV CBS System

The majority of healthcare workers, 31/34 (86%), reported that either multidisciplinary team (MDT) or rapid response team (RRT) met and discussed HIV CBS data as an agenda while 23/34 (68%) reported that data were analyzed and utilized at the facility level. The number of public health actions taken after the analysis report was 21/34 (62%). Data were also used to identify new HIV acquisition hotspots for community interventions 30/34 (83%) (Table 4).

## 6. Discussion

We evaluated the HIV CBS system to assess whether the objectives of the system were being met, its usefulness, the extent of underreporting, and the reasons for underreporting of newly diagnosed patients in the REDCap database. We found that the major reason for underreporting HIV cases through the REDCap database was due to work overload. Health workers in non‐antiretroviral treatment (ART) service units were not willing to complete the case reporting form, while health workers in ART service units reported low confidence in completing the forms within the required time, resulting in underreporting of cases. Educating health workers on reporting requirements reduces their perception of HIV case reporting as burdensome. Other reasons contributing to underreporting of HIV cases through the REDCap system included high staff turnover, limited competence in using the REDCap platform, and inadequate feedback from central levels, all of which may have negatively affected the consistency and overall performance of the reporting system [9].

### 6.1. Stability

The role of feedback cannot be overemphasized in coordinating surveillance activities, increasing awareness, or reinforcing the importance of participating in the HIV CBS system. The system was not stable, as electric power outages and software upgrades affected the system in some facilities. Seven facilities lacked functional generators for power backup, and facilities with functional generators faced fuel shortages. Our findings were consistent with those that found that some of the challenges in the HIV CBS system were electricity outages and a lack of supplies to operate the system [3, 12]. An extensive backup plan was not in place, as the HIV CBS case reporting forms that could be used as backup paper‐based records were not available at the four facilities.

### 6.2. Representativeness

We found that the overall representativeness of the HIV CBS system was poor. These findings differ from those reported by Gortakowski et al. (2010), who evaluated an HIV surveillance system in New York City (NYC). The NYC system had long‐established electronic reporting and standardized data entry procedures that enabled timely, complete, and representative reporting. In contrast, the HIV CBS system in our setting is relatively new, and electronic mobile devices became available only after 2020, resulting in substantial backdated data entry. These system differences likely explain the discrepancy in representativeness between the two studies [13].

Only three private facilities participated in the system, which further limited representativeness. However, the distribution of community self‐testing kits by partner agencies facilitated the inclusion of individuals regardless of age, sex, or locality. This aligns with findings from Okeafor et al. in Nigeria, where representativeness was similarly constrained by the limited involvement of private health facilities [14].

### 6.3. Timeliness

We found that the timeliness of the HIV CBS system was 92%, as HIV CBS case reporting forms were entered daily by the majority of healthcare workers, and there was real‐time automatic transmission of data to the national level. This is consistent with the findings of Ezeudu et al. (2016), who evaluated a similar HIV CBS system in Enugu State, Nigeria. The Nigerian system also used routine daily data entry and electronic transmission of case reports, although it operated within a more established electronic reporting framework compared to our setting. These similarities in system structure and data‐flow processes likely explain the comparable timeliness observed in both studies [15].

### 6.4. Completeness

Data quality was measured using completeness and validity. We found that the data quality of the HIV CBS system is good. Completeness was high for client identifier information; however, it was low for the WHO clinical staging. These findings are consistent with those of Naqibullah et al. (2020), who evaluated Afghanistan’s HIV surveillance system, which also relies on routine case‐based reporting from health facilities and uses standardized case reporting forms similar to our system. In Afghanistan, demographic variables showed high completeness, while clinical variables (screening, diagnosis, and treatment) had lower completeness [16]. The similarities in reporting structure and reliance on manual or partially electronic systems likely explain the comparable patterns of data completeness.

### 6.5. Usefulness

This reinforces the importance of HIV CBS, as these shortcomings in data quality affect the usefulness of data for programming. We found that HIV CBS to be simple when using the HIV CBS case reporting forms. However, the notification of HIV cases through the CBS system was not simple, as the majority of healthcare workers could only log in and creates a REDCap, which ultimately required more time than when using the paper‐based HIV CBS case reporting form. Several gaps were observed in terms of the retrieval and editing of patient records, generation of reports, and analysis of results.

This is consistent with the findings of Sukums et al. (2014), who concluded that given the low levels of computer knowledge among rural health workers in Africa, it is important to provide adequate training and support to ensure the successful uptake of electronic systems in primary health facilities in Burkina Faso, Ghana, and Tanzania [17, 18].

Training ensures that users are competent and comfortable with the use of the new CBS system, and reassessment status should be ascertained to evaluate the training gaps. The system was found to be flexible, consistent with findings by Okeafor et al., who found the HIV CBS system to be flexible in Rivers State, Nigeria [13].

The HIV CBS system could also be integrated with several reporting systems; however, the use of parallel HIV reporting systems, including paper‐based systems (HIV CBS case reporting forms and registers), EPMSs, and DHIS‐2 systems, results in the duplication of tasks. This is consistent with the findings of Ogungbemi et al. (2012), who found that the use of multiple unlinked HIV databases to capture program monitoring data results in duplication of effort and poor resource use [19].

### 6.6. Acceptability

We found that the HIV CBS system was acceptable, as all healthcare workers expressed willingness to continue participating in the system because it presented opportunities for reduced paperwork, secure storage of records, and simple report generation. Our findings are consistent with those of Okeafor et al., 2017, where an evaluation of the HIV CBS system in Rivers State, Nigeria, revealed that all stakeholders were willing to continue to participate in the surveillance system [14].

The majority of the healthcare workers had good knowledge of the HIV CBS system. This may be because health workers play a central role in HIV interventions, which enhances their knowledge of the HIV CBS system. Data were analyzed at the facility level, and the majority of healthcare workers found the system useful for their daily operations. Data from the HIV CBS system was used to identify hotspots for targeted interventions, to identify risky behavior for communication strategies, drugs, and HIV test kit supply management, and to identify challenges. This is consistent with Nsubuga et al., 2020, who found that the system was utilized at the facility level to analyze client data and enable staff to accurately order the exact number of antiretroviral drugs for their clients [13].

### 6.7. Limitation of the Study

Although we assessed data quality by reviewing sampled HIV CBS case reporting forms for completeness, accuracy, and consistency, we were unable to evaluate the broader data quality assurance processes of the system. Documentation related to training, mentorship, supportive supervision, and feedback mechanisms were not available at most facilities. As a result, our assessment of system‐level data quality relied primarily on interviews with healthcare workers, which may have introduced recall bias and social desirability bias. Additionally, because gaps in institutional data quality practices could not be independently verified, some aspects of CBS system performance may have been under‐ or overestimated.

## 7. Conclusions

We concluded that the overall knowledge levels of health workers regarding the HIV CBS system were satisfactory. The HIV CBS system with recency testing performed well in terms of timeliness, flexibility, and acceptability with high data quality; however, representativeness, stability, and simplicity were not satisfactory. The reasons for underreporting of HIV cases through the CBS system are mainly work overload, high staff turnover, inadequate REDCap database competence, and limited feedback from higher levels.

There were no SOPs for backup operations during system interruptions, and health workers required further training on the use of the CBS system. The system was useful because the information was used to manage stocks and identify hotspots of new HIV acquisitions.

## 8. Recommendations

### 8.1. To the Health Facility

• We also recommend the distribution of WHO clinical staging guidelines for HIV, the servicing of generators for backup electricity, and the installation of solar power.

### 8.2. To the Regional Health Bureau

• We recommend on‐the‐job refresher training on the CBS system for healthcare workers and data entry clerks to address identified training gaps as well as data analysis training.
• Support and supervision visits at higher levels and technical staff are critical.

**Nomenclature**
CBS: Case‐based surveillance
CDC: Centers for Disease Control and Prevention
COVID‐19: Coronavirus disease
CRF: Case report form
CSA: Central Statistical Agency
CPAC: CBS Publication Advisory Committee
DHIS‐2: District Health Information System
EFY: Ethiopian Fiscal Year
EPHI: Ethiopian Public Health Institute
FCH: Family and Child Health
HF: Health facility
HIV: Human immunodeficiency virus
HTS: HIV testing services
VMMC: Voluntary Male Medical Circumcision
REDCap: Research Electronic Data Capture
RHB: Regional Health Bureau
WHO: World Health Organization
ZHD: Zonal Health Department
IRB: Institutional Review Board

## Ethics Statement

Ethical clearance was obtained from the Ethiopian Public Health Institute (EPHI) CBS Publication Advisory Committee (CPAC) and the EPHI Institutional Review Board (IRB). The letter of support was obtained from the EPHI HIV/TB Directorate and Dire Dawa City Health Bureau and 14 HIV CBS‐implemented health facilities. Written informed consent was obtained from all study participants to ensure their voluntary participation and confidentiality. Participants’ anonymity was maintained throughout the study by not using their names or addresses. Additionally, COVID‐19 infection prevention and control practices were observed during the interviews to prioritize participant safety. To uphold data confidentiality and security, the research team adhered to the national CBS confidentiality agreement by signing it before accessing any data. During all phases, all methods carried out in the study were performed in accordance with the relevant guidelines and regulations.

## Consent

Please see the Ethics Statement.

## Disclosure

An earlier version of this manuscript was published as a preprint on Research Square: https://www.researchsquare.com/article/rs-3281831/v1 [20]. All the authors agreed to be accountable for all aspects of the work in ensuring that questions related to the accuracy or integrity of any part of the work are appropriately investigated and resolved. All authors have approved the final manuscript.

## Conflicts of Interest

The authors declare no conflicts of interest.

## Author Contributions

Fitsum Hagos, the corresponding author, was the major contributor to preparing the manuscript. Fitsum Hagos, Aklesiya Kassahun, and Ashenafi Sisay supported the analysis and interpretation of the data and revised the manuscript critically for important intellectual contents. Additionally, it contributed substantially to the design of the study and critical revision of the final approval of the manuscript to be published. Ashenafi Sisay and Aklesiya Kassahun contributed a lot to the conception, revision, and approval of the final version of the manuscript.

## Funding

No funding was received for this research.

## Data Availability

The datasets used and/or analyzed during the current study are available from the corresponding author on reasonable request.
